# No water, no mating: Connecting dots from behaviour to pathways

**DOI:** 10.1371/journal.pone.0252920

**Published:** 2021-06-10

**Authors:** Homica Arya, Regan Toltesi, Michelle Eng, Divita Garg, Thomas J. S. Merritt, Subhash Rajpurohit

**Affiliations:** 1 Division of Biological and Life Sciences, School of Arts and Sciences, Ahmedabad University, Navrangpura, Ahmedabad, Gujarat, India; 2 Department of Chemistry and Biochemistry, Laurentian University, Sudbury, Ontario, Canada; Biomedical Sciences Research Center Alexander Fleming, GREECE

## Abstract

Insects hold considerable ecological and agricultural importance making it vital to understand the factors impacting their reproductive output. Environmental stressors are examples of such factors which have a substantial and significant influence on insect reproductive fitness. Insects are also ectothermic and small in size which makes them even more susceptible to environmental stresses. The present study assesses the consequence of desiccation on the mating latency and copulations duration in tropical *Drosophila melanogaster*. We tested flies for these reproductive behavioral parameters at varying body water levels and with whole metabolome analysis in order to gain a further understanding of the physiological response to desiccation. Our results showed that the duration of desiccation is positively correlated with mating latency and mating failure, while having no influence on the copulation duration. The metabolomic analysis revealed three biological pathways highly affected by desiccation: starch and sucrose metabolism, galactose metabolism, and phenylalanine, tyrosine and tryptophan biosynthesis. These results are consistent with carbohydrate metabolism providing an energy source in desiccated flies and also suggests that the phenylalanine biosynthesis pathway plays a role in the reproductive fitness of the flies. Desiccation is a common issue with smaller insects, like *Drosophila* and other tropical insects, and our findings indicate that this lack of ambient water can immediately and drastically affect the insect reproductive behaviour, which becomes more crucial because of unpredictable and dynamic weather conditions.

## 1. Introduction

The impact of climate change on flora and fauna are visible across the globe now [1,2] and ectotherms are particularly at greater risk [3,4]. Insects are specifically well suited for climate change impact studies as they have been reported as effective bio-indicators, have wider presence and are easily affected by ambient climatic conditions [5]. Environmental disturbances often result in significant changes in insect physiology and behaviour [6,7]. Both survival and reproduction have been shown to be greatly affected in insects by environmental stresses and drought is one of the key ecological factors which dictates species distribution and survival [8–12].

Availability of water and environmental temperature are undoubtedly the two most pivotal abiotic variables responsible for the abundance and distribution of organisms on this planet [13,14]. Under natural conditions the interaction of these two environmental variables have also been found involved in dictating reproductive output of insects [15]. The tropics, especially recently, are experiencing unexpected and extensive seasonal variation [16]. The effects of such environmental variation on insect reproduction remains largely unexplored, although effects of changes in humidity and temperature have been explored [17–19]. As a group, insects are especially sensitive to water-related challenges because of their small body sizes and consequently large surface area to volume ratio [20–23]. Survival and reproduction are essential components of an organism’s fitness, but both are energetically exhaustive [24]. Therefore, survival-mating trade-offs are frequently observed in organisms under unfavorable or harsh situations, with organisms trading one for the other by a differential allocation of time and resources [25]. The assumed explanation is that mechanisms involved in survival and reproduction compete for resources that are limited [26]. Especially at milder stress levels, increased desiccation resistance is known to come at the cost of lower copulation success [9]. Under ecologically harsh conditions such as the absence of food or suitable reproductive sites, female flies may consider sparing resources for survival over breeding [12]. Equivalently, favorable circumstances can enhance reproductive ability, as is shown by the increased copulation success in the presence of nutritional sources, such as yeast and sugar [27].

The courtship practices of *Drosophila melanogaster* are complex, well-described and highly sensitive to environmental stressors, making this species a strong model for assessing influences of both internal and external stresses on reproduction [8,11,28–30]. Conventional courtship rituals performed by male flies include female following, subsequent specific orientation to females, song production via vibrations of a perpendicularly extended wing, licking of female genitalia and, conclusively, attempts at mounting, which may or may not result in successful copulation [31,32]. Alterations in mating behaviour and reproduction can be quantified by measuring a suite of parameters, including courtship duration, courtship song analysis, copulation success, mating latency and mating duration.

The effect of several environmental stressors on *D*. *melanogaster* reproduction has already been quantified. For example, courtship duration is lengthened under chronic exposure to dim light during the night by rapid cold hardening and the effect of starvation stress [8,11,29]. Lines selected for resistance to cold stress also exhibit reduced mating latency and increased mating frequency and fecundity [33]. Further, larval crowding results in increased male courtship frequency [30]. Additionally, starvation stress leads to reduction of the rate of wing bouts, an essential part of the male courtship behaviour, performed by the male *Drosophila* [29]. Developmental temperature also influences fecundity and egg to adult viability with a maximum of viable offspring at the optimal temperatures [34]. Additionally, short‐term exposure to higher temperatures greatly reduces adult survival, mating frequency and female fecundity [35]. On the whole, most studies support the survival-reproduction trade-off hypothesis. However, the possible effects of depleting body water levels on reproductive behaviour and possible trade-offs have not yet been quantified. In this study, the effect of dehydration on the mating success, mating latency, copulation duration and changes in whole metabolome of *D*. *melanogaster* have been assessed.

In order to gain a further understanding of how desiccation affects reproductive ability, an untargeted metabolomic analysis was performed. Desiccation would undeniably put stress on the metabolic function of the flies and the pathways that are affected may play a role in the survival-reproduction trade-off being studied. Alterations in carbohydrate and/or triglyceride levels were expected in order to increase water availability [36–38]. Phenylalanine and tyrosine have been found to play a large role in the reproductive fitness of insect *R*. *prolixus* [39], at least three species of mosquitos [40–42] and the olive fruit fly, *Bactrocera oleae* [43]. Therefore, we expected to see an impact on the pathways involving carbohydrates and/or triglycerides as a result of the desiccation. Furthermore, we proposed that there would be changes in the phenylalanine and tyrosine pathway, as desiccation is hypothesized to have a negative impact on the flies’ reproductive fitness.

## 2. Materials and methods

### 2.1. Fly collection and maintenance

Flies were collected from a tropical highland location in western Himalayas (Rohru, Himachal Pradesh, India; 31.2046°N, 77.7524°E, Altitude 1554 meters) using banana baits. From this collection, 100+ isofemale lines were established and species identification of these lines was confirmed based on the F1 male phenotypic characteristics. All the lines were maintained in the laboratory on standard media (Agar–Jaggery–Yeast–Maize flour) at 25°C ambient temperature, with a 10:14 hours light: dark cycle. A total of 40 *D*. *melanogaster* lines were chosen at random for this study.

### 2.2. Outcrossing of isofemale lines

We established 6 replicate laboratory populations (A1, A2, A3, A4, A5, and A6) by outcrossing *Drosophila melanogaster* isolines for 10 generations. For outcrossing, 10 males and 10 females, each, from 40 randomly chosen isofemale lines (800 adults: 400 males + 400 females) were pooled in a 12*6*6 inch dimension plexiglass cage.

For experimental work, flies were reared in vials in a density-controlled manner, with the egg count maintained at approximately 30 eggs per vial and incubation in a BOD Incubator set at 25°C with a 10:14 hours light: dark cycle. Virgin males and females were subsequently collected through standard fly procedures (i.e. adults were collected within first two hours post-eclosion) and aged for three–four days before subjecting them to assays.

### 2.3. Desiccation assay and mating chambers

Three to four day old virgin males and females were desiccated (without food and water) individually in narrow *Drosophila* vials. Dry conditions in vials were generated using Silica gel (Sigma-Aldrich, USA), along with a foam plug to prevent direct contact of the Silica gel with the flies. Vials were sealed using parafilm. Silica gel brings down humidity to less than 2 percent in a sealed tube. Six replicate groups were used for each set of desiccation exposure ranging from 0 (control) up to 16 hours. Eighteen flies (i.e. nine pairs) were assessed for each duration per replicate with a sum total of 162 flies per replicate and an overall count of 486 pairs. After solitary desiccation, flies were transferred to mating chambers (in-house design) such that each well contained a male and a female. For controls, the flies were directly transferred from the resident vials to the chambers. Until all the treatment flies were loaded, males and females were kept separated by the teeth (window panel) of a movable comb. The comb was removed at the commencement of the assay allowing interaction between males and females. All experiments were strictly carried out in the morning hours (09.00–12.00) using manual aspiration (i.e., without CO_2_) to relocate flies in order to avoid effects arising from anesthetics. All trials were conducted at an ambient temperature of 25°C and the assays were recorded using a video camera (HMX-F90BN/XAA, Samsung). Subsequently, video recordings were processed for the time measurements of various mating parameters (mating success, latency and duration).

### 2.4. Mating assays

In the first round of assays, flies were desiccated for 0 (control), 2, 4, 6, 8, 10, 12, 14 and 16 hours, in order to establish the effect that dehydration imparts on the mating behaviour (success, latency and duration) of these flies. Mating was considered successful if the couple remained attached in the mating posture for more than five minutes.

### 2.5. Water content measurement

For every replicate, 30 individuals per sex were individually subjected to desiccation followed by pooling flies in 0.2 cubic centimeter glass tubes with five flies per tube. Similarly, control tubes without the dehydration treatment were set-up. After storing flies in airtight 0.5 mL tubes at -20°C for 24 hours, they were first weighed for their wet weight and then re-weighed after a 24-hour incubation in an oven at 50°C, to obtain the dry weight. Subsequently, water content per fly was calculated by subtracting the dry weight from the original (wet) weight of the flies. All measurements were recorded using an Ohaus PAG214 Analytical Balance with resolution of 0.0001mg (Ohaus Instruments Co. Ltd. Shanghai, China).

### 2.6. MT_50_ value

The time of desiccation at which 50% of flies do not participate in mating events is designated as the MT_50_ value, which was calculated using logit regression (Fig 3 which corresponds to the desiccation duration that results in lowering of mating success by 50% (or MT_50_ value) as compared to the controls)). The MT50 value was found to be 7 hours and 39 minutes based on the linear regression equation (MT50 value; using logit regression; Fig 3). Following experiments compares controls vs MT50 hr treated populations.

### 2.7. Metabolomics

In order to assess the metabolome profile, 60 flies per sex per replicate were flash frozen in two sets, one directly aspirated from the resident vials (controls) and one after the desiccation treatment (experimental). We accessed the effects of desiccation on the broad metabolome by desiccating flies for (MT_50_ value; 7 hours and 39 minutes; see section 2.6) hours and comparing their metabolome to that of control flies. We exposed males and females separately to experimental and control conditions and then directly transferred them to liquid nitrogen. This protocol resulted in four tubes per replicate cage and a total of 24 samples (12 control and 12 experimental; see S1 Fig). Each sample was then homogenized in a volume of homogenization buffer standardized to the weight of the sample. Each of the 24 samples were weighed to the nearest 0.01 mg using a Mettler Toledo microbalance MX5. To create a large enough sample volume for analyses, a volume, in μL, of 85:15 of ACN:H2O solution equal to eight times this weight was added to each sample, in a random order. Metabolites were extracted from whole flies by homogenizing samples in a mixer mill (TissueLyser, Qiagen) using 3.5 mm stainless steel beads in 1.5 mL microcentrifuge tubes at 30 Hz for 2 minutes. After homogenization, the samples were centrifuged for 20 minutes and the supernatant was transferred to new screw-cap 1.5 mL tubes. The samples were then chilled in a -20°C freezer for one hour to precipitate proteins and then centrifuged twice to make sure there were no large particles, with the solutions transferred to a new tube after each centrifuge. The samples were then transferred to LCMS tubes, ensuring minimal movement so as to not re-suspend the particulate matter during transfers. Five microliters of each sample were taken and pooled to make quality control samples. Finally, all samples were labeled and frozen in liquid nitrogen in a -20°C freezer until analysis.

We performed a positive mode, untargeted, approach for metabolite analysis using a Water’s Acquity Class I Ultra-High Performance Liquid Chromatography Xevo G2-XS Quadrupole Time of Flight Mass Spectrometer (Water’s UPLC-Qtof-MS). We used an Acquity BEH Amide 1.7μm 2.1x150mm column with a column temperature of 40°C. A column guard was used to filter out larger particles that may have been missed. A calibration was performed with a solution of sodium formate; and samples only run when the RMS was less than 1 ppm. The solvents were prepared as mobile phase A and mobile phase B. Mobile phase A consisted of a 20 mM ammonium formate in LCMS H2O solution and mobile phase B consisted of a 0.1% formic acid in acetonitrile solution. A 15:85 mobile phase A to mobile phase B solution was used. 2 μL of each sample was run in a positive acidic mode with a flow rate of 0.4 mL/min and a run time of 18 minutes per sample.

The data produced by the LCMS was analyzed using Progenesis QI by Nonlinear Dynamics (a Water’s company), which is a small molecule discovery analysis software for LCMS data. The workflow of this software includes importing data, aligning the sample runs, peak picking, identifying and reviewing compounds and compound statistics. We performed pathway enrichment analysis using Metaboanalyst (www.metaboanalyst.ca) which utilizes the *mummichog* software [44]. The pathway enrichment analysis approach matches all compounds using the Kyoto Encyclopedia of Genes and Genomes (KEGG) database and uses *D*. *melanogaster* as the reference genome [45]. Using an error of 5ppm when matching metabolites, all compounds were analyzed on Metaboanalyst. A value of p < 0.05 was used as a cut-off to determine which metabolites were considered significantly different between the control and experimental groups.

### 2.8. Statistical analyses

Trait variability between populations was analysed through ANOVA. For all the traits, replicate population’s means s.e. were used for illustrations and tabular data. Correlations between different traits were calculated for replicate populations. Statistical calculations and illustrations were performed with the help of Statistica_TM_ 11.0.

## 3. Results

### 3.1. How does desiccation affect mating success mating latency?

**Fig 1:**
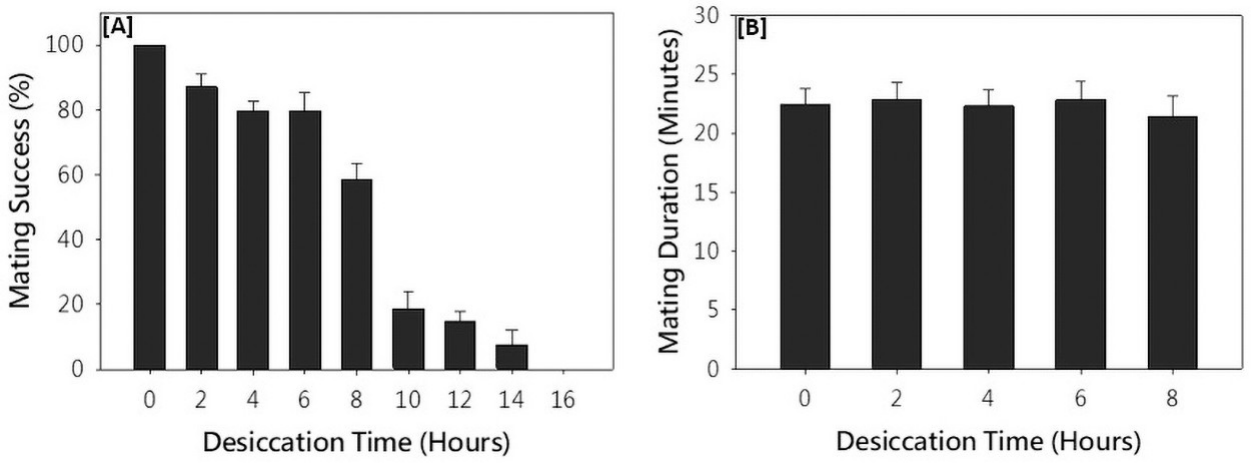
Mating success and mating duration of *D*. *melanogaster* adults under varying desiccation treatments. A. Desiccation Treatment and Mating Success. Bar graph representing the effect of desiccation time on the mating success of outcrossed *D*. *melanogaster* flies. Each bar represents the average value recorded for a total of 54 pairs (9 pairs per replicate) and the error bars depict values of standard error. P-value obtained from ANOVA: Single Factor test: 1.4*10^−20^. B. Desiccation Time and Mating Duration. Bar graph illustrating the effect of desiccation on the mating duration (i.e. copulation duration) of outcrossed *D*. *melanogaster* flies. Each bar is the average value recorded for 54 pairs, the bars delineating standard error. P-value obtained from ANOVA: Single Factor test: 0.69034.

There was a negative correlation between desiccation duration and mating success (r = -0.96) Fig 1A)). With every two hour increase in desiccation treatment, the number of pairs that mated in a particular trial is seen to decline, ultimately concluding in absolute refrainment from copulation (16 hr) ANOVA *P* = 1.4*10^−20^; *d*.*f*. 53; F: 54.289)).

**Fig 2:**
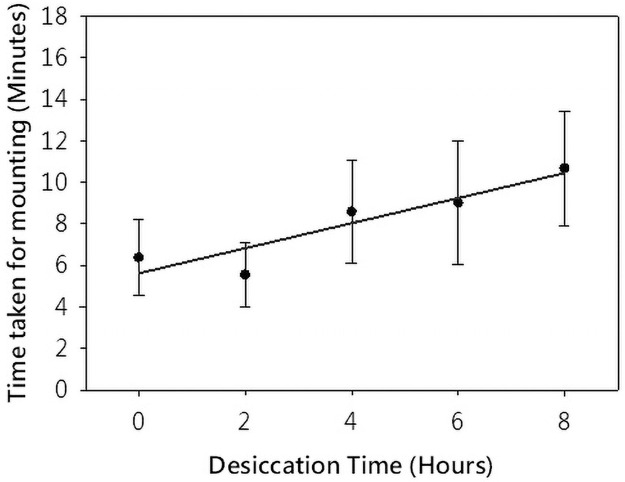
Desiccation time and mating latency. Desiccation time positively affected the time taken for mounting i.e. mating latency. Each data point denotes the average value recorded for 54 pairs, with the bars representing standard error values. P-value as per ANOVA: Single Factor test: 0.0024.

Mating latency, i.e., time taken for the successful mounting of a male on a female after contact between the two is permitted, increased in parallel with the desiccation duration (Fig 2; ANOVA *P* = 0.0024; *d*.*f*. 29; F: 5.557). Although assayed up to 16 hours, the presented data for mating latency represents values up to 8-hour dessication experiments since the mating success (pairs that indulged in mating) went considerably down post that timepoint thereby causing a stark reduction in the number of replicates.

### 3.2. MT_50_ value determination, copulation duration, and water loss quantification

**Fig 3:**
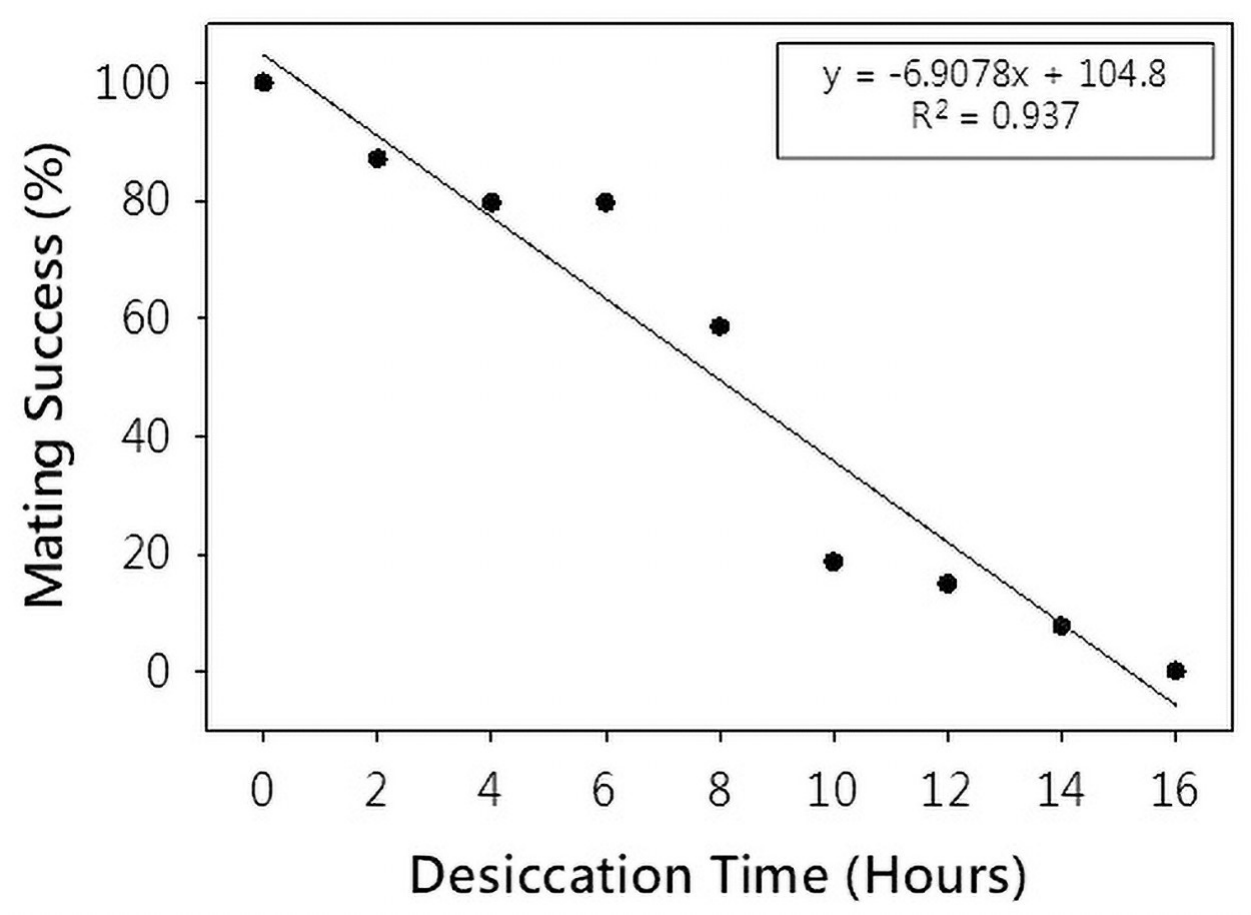
MT_50_ value estimation. Scatter plot of mating success versus desiccation duration with inset: linear equation and R-squared value. The MT_50_ value (desiccation time corresponding to 50% mating success) thus obtained was 7 hours and 39 minutes (Logit Regression). Each data point represents the average value recorded for a total of 54 pairs (9 pairs per replicate) and the error bars depict values of standard error. P-value obtained from ANOVA: Single Factor test: 1.4*10^−20^.

Desiccation increased the percentage of females that did not participate in mating events. The time at which 50% of flies do not participate in mating events is designated as MT_50_ value. MT_50_ value was calculated using Fig 3 which corresponds to the desiccation duration that results in lowering of mating success by 50% as compared to the controls. The MT_50_ value was found to be 7 hours and 39 minutes based on the linear regression equation (MT_50_ value; using logit regression; Fig 3).

Once pairs were formed, no difference was observed in mating duration across desiccation treatment hours (Fig 1B; ANOVA *P* = 0.69; *d*.*f*. 29; F: 0.565).

**Fig 4:**
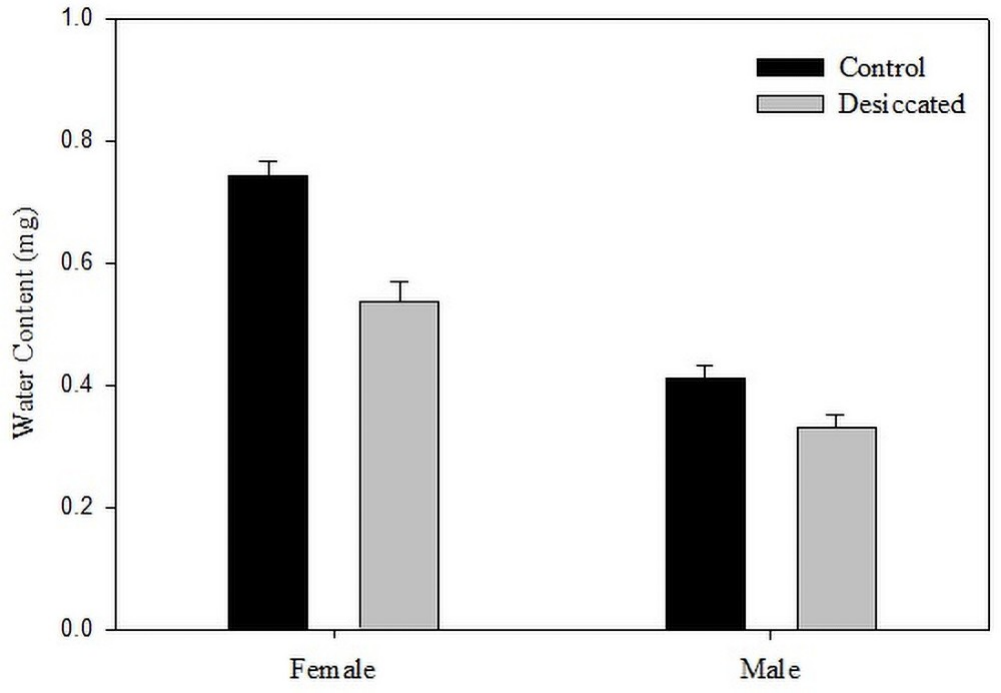
Water content measurement. Bar graphs depicting water content as measured in untreated flies, in comparison with desiccated flies (Treatment time = 7 hours 39 minutes). Each bar denotes the average value recorded for 180 flies (36 groups of 5) and the error bars represent values of standard error.

To quantify the amount of water lost during the process of desiccation, the water content of flies was compared to that of flies desiccated for 7 hours and 39 minutes (Fig 4). Females exhibited an average water loss of 0.21 mg (29%) whereas water content in males was shown to reduce by 0.08 mg (19%), both combinatorically leading to a 50% decrease in mating success.

### 3.3. Metabolomics

Assays on mating success clearly demonstrated that flies avoided mating when they were desiccated. Energy reserves (e.g. carbohydrates, proteins, and lipids) and their role in desiccation tolerance in flies have been explored at length but pathways involved in this particular period (until MT50) were never investigated. We expected to see changes in metabolite shift and pathways involving signaling relating to mating behavior. We detected a total of 23,791 compound ions, of which 14,962 compounds had a fold change > 2 (63% of total). A total of 4,154 compounds (17% of total) were significantly different, p < 0.05, between the experimental and control flies (Table 1 and S1 Table). 60 metabolic pathways were found to have at least one significant metabolite hit (at least one pathway member was significantly different between control and desiccated flies), with a total of 234 metabolite hits that were significantly different between the control and experimental groups.

**Table 1:** An overview of the detected compounds from the LCMS data.

Filter	Number of Compounds	% of Total
Fold change > 2	14,962	63
p < 0.05	4,154	17
Fold change > 2, p < 0.05	3,865	16

**Fig 5:**
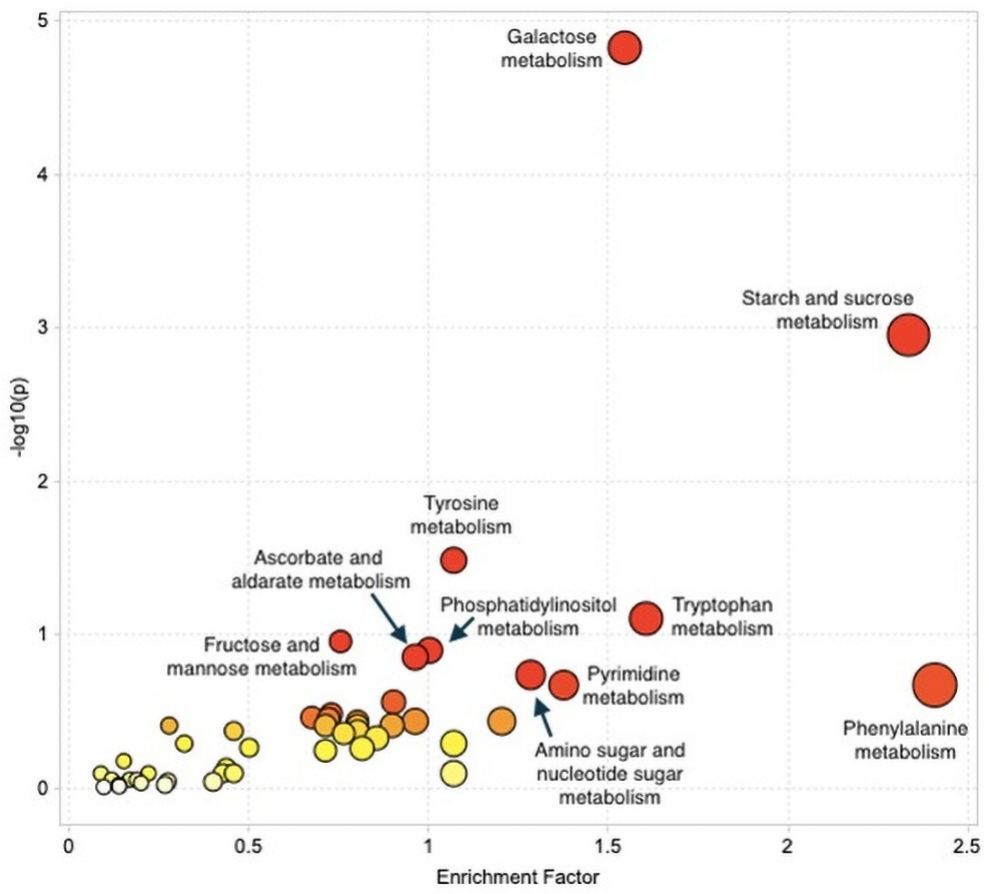
Metabolomics results. The pathways determined to be most changed between the treatment and control group are galactose metabolism, starch and sucrose metabolism, and phenylalanine metabolism. Other pathways of note are tyrosine metabolism and tryptophan metabolism. With increasing–log10(p) value (along the y axis), the colour changes from white to red. With increasing enrichment factor (along the x axis), the diameter of the circle increases.

To determine the pathways that were most changed between the control and experimental groups, the p value (significance) of the pathway and the enrichment factor of the pathway were plotted, as depicted in Fig 5. As the enrichment factor increases, the colour of the pathway circle turns from white to red; as the -log10(p) increases (hence, as p value decreases) the diameter of the pathway circle also increases. The enrichment factor is the ratio between the number of significant metabolite hits from the data and the expected number of hits just by chance within a certain pathway [46] and is calculated using Fisher’s exact test (FET) [44]. This calculation is done to normalize the results since the number of metabolites within a given pathway varies. As seen in Fig 5, three pathways are distinctly separate from the rest of the group due to high enrichment factor and -log10(p) values, making them of interest for further investigation. Relative to the other metabolic pathways, starch and sucrose metabolism, galactose metabolism, and phenylalanine, tyrosine, and tryptophan biosynthesis were the most changed pathways between the control and experimental groups (Table 2 and S2 Table). While changes in metabolites were expected, especially around sugar metabolism, these specific results are novel, especially the amino acid biosynthesis pathways, and although exploratory suggest interesting avenues for future research and testing.

**Table 2:** Top significantly changed pathways based on their calculated enrichment factor and significance value.

Pathway name	Pathway metabolites	Sig. hits	Exp. hits	Enrichment factor	FET p value
Starch and sucrose metabolism	11	11	2.3065	4.769	6.2E-5
Galactose metabolism	27	25	5.6613	4.416	2.4E-9
Phenylalanine, tyrosine and tryptophan biosynthesis	4	3	0.8387	3.577	0.203

## 4. Discussion

This study evaluates the influence of the environmental stress of desiccation on the mating behaviour of tropical *D*. *melanogaster*. Our most striking finding is a negative correlation between desiccation time and mating success. Dehydration of flies did not bring about any change in the copulation duration, however it resulted in an increased latency of mating, indirectly indicative of longer courtship durations as was observed in the video trials, suggesting that dehydrated females take a longer time to accept dehydrated males.

Experimental analysis revealed that the duration of desiccation is positively correlated with mating latency and mating failure, while having no influence on the copulation duration (Figs 1A, 1B and 2). Gefen and Gibbs [9] examined the mating success in laboratry selected populations for desiccation and their controls and reached to similar conclusions. Our work on tropical populations of the same species established that this response is evolutionarily conserved. The populations used by Gefen and Gibbs [9] were of temperate origin. These results are also consistent with the survival-reproduction trade-offs that have been frequently reported [12,27,47–50]. Copulation is energetically exhaustive but is fundamental to the continuation of a genetic lineage through evolutionary time, so succesful mate and mating choices are crucial to evolutionary survival [51]. Whether natural humidity patterns are strong enough to impose such changes in mating behaviour is area of further investigation [52]. This needs to be tested under natural conditions and across geographical areas.

Pathway enrichment analysis is an effective way to provide an overall representation of the large amount of data including the large number of individual metabolites that untargeted metabolomic analysis creates [53]. In our experiment, three metabolic pathways were found to be highly altered between the control and desiccated fly groups: starch and sucrose metabolism, galactose metabolism, and phenylalanine, tyrosine and tryptophan biosynthesis (Fig 5).

*Drosophila* rely heavily on carbohydrate, i.e. starch and sucrose metabolism during desiccation. Fasted *Drosophila* flies have lower glycogen levels compared to non-fasted flies [54] and some earlier work has shown a reduction in triglycerides under desiccation [38]. However, emphasis has been placed on flies relying primarily on carbohydrate metabolism as an energy source when desiccated, with a seven-fold increase in the rate of consumption relative to starvation [37]. Although lipids provide over twice as much energy per gram as carbohydrates (which would be favorable under starvation conditions), glycogen provides more water per gram than lipids; therefore, the total amount of water available after metabolism is much higher in carbohydrates than in lipids [36]. Given this relationship, it is reasonable that flies seem to rely primarily on carbohydrate metabolism when desiccated in order to increase water availability. Our results support this idea, showing that starch and sucrose metabolism and galactose metabolism pathways differ significantly (p < 0.001) in the desiccated group and exhibit no apparent difference in lipid metabolism between the control and desiccation groups.

Galactose metabolism may tie into desiccation response in flies through the metabolite’s connection to trehalose. Trehalose is a non-reducing disaccharide found in *Drosophila* (as well as other insects and organisms), that is both a blood sugar and protectant whose production is induced by stress conditions such as heat, desiccation, oxidative stress, and other adverse conditions [55,56]. The main source of trehalose in *Drosophila* comes from diet carbohydrates, specifically UDP-glucose and glucose-6P [56]. The main source of UDP-glucose for trehalose synthesis is from galactose metabolism [57]. Other studies [58] have demonstrated the importance of trehalose and trehalose-synthesizing enzymes in dietary stress response and our finding that the galactose metabolism pathway is substantially altered in the desiccated flies may reflect the increase in production of trehalose as part of a stress response.

Phenylalanine, tyrosine, and tryptophan biosynthesis was also significantly altered in the desiccated group. Phenylalanine and tryptophan are essential amino acids for all insects and must be ingested in the diet, while tyrosine is synthesized from phenylalanine via the enzyme phenylalanine hydroxylase (PAH) [43]. Therefore, under periods of starvation and desiccation, all three amino acids will have limited availability. Reduction or loss of PAH activity negatively affects the reproductive fitness of the insect *R*. *prolixus* [39] and at least three species of mosquitos [40–42]. Similarly, the addition of phenylalanine and tyrosine analogues, which induces the dietary deletion of the amino acids, significantly reduces fecundity and egg hatchability in the olive fruit fly, *Bactrocera oleae* [43]. These findings demonstrate the critical role of these amino acids in insect egg production and viability and broadly in development and reproduction. The alteration in the phenylalanine, tyrosine and tryptophan biosynthesis pathway that we observe in desiccated *Drosophila melanogaster* may reflect these essential roles and suggest a connection between them and changes in copulation inclusive of reduction in mating success.

## 5. Conclusions

This study demonstrates vital correlations between dehydration and reproduction and highlights the immediate negative consequences that environmental stressors can bring about on fly populations (across tropical and temperate regions). It would be interesting to assess other species on a similar scale to determine how broadly consistent these patterns are across insects and invertebrates. Such broad trends would be an alarming cause for concern, because of the ecological imbalances these could result in given the ecological importance of insect communities and the present unpredictable, and ever-increasing environmental disturbances.

## Supporting information

S1 Fig Experimental design of metabolomics analysis. Control and MT_50_ flies were immediately preserved in liquid nitrogen and processed for metabolome analysis.
(JPG)

S1 Table Metabolome compound hits data. (CSV)

S2 Table Pathway enrichment analysis data. (CSV)
