# Supplementary figures and images for: No water, no mating: Connecting dots from behaviour to pathways
Source: PLoS One. 2021 Jun 10;16(6):e0252920. doi: 10.1371/journal.pone.0252920 (PMC8192009; doi:10.1371/journal.pone.0252920)

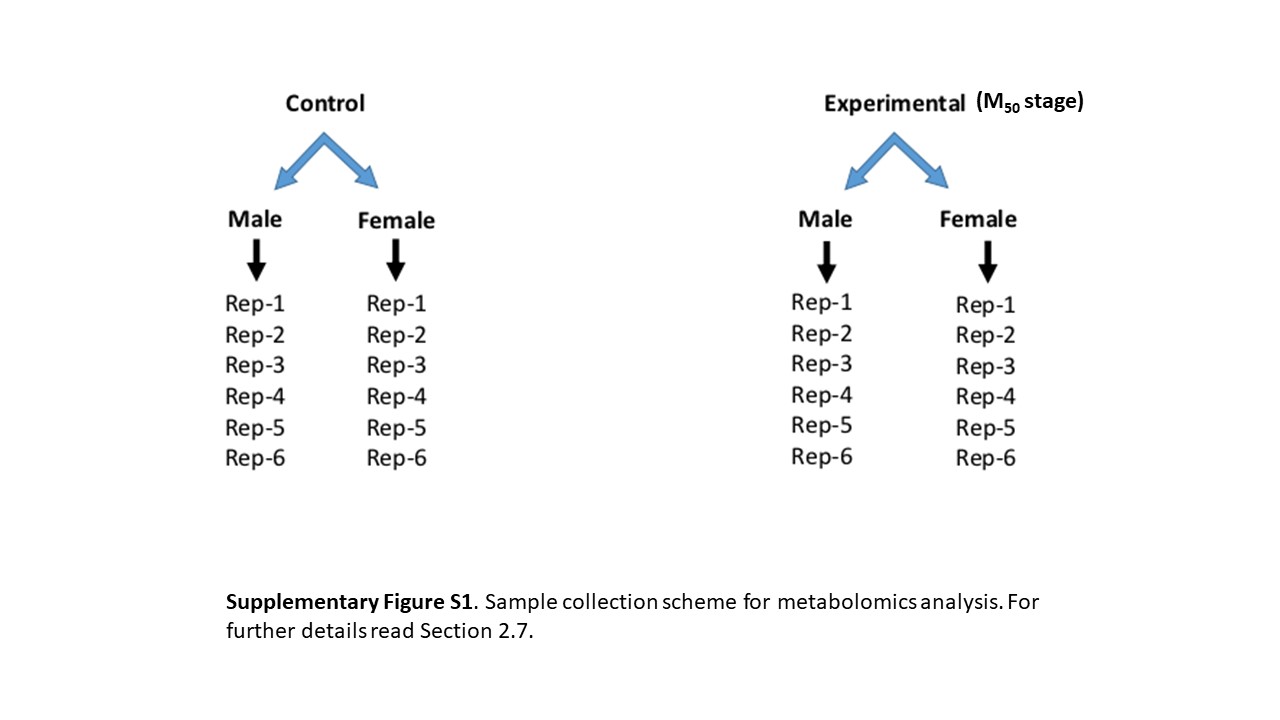

Supplement: S1 Fig — Control and MT50 flies were immediately preserved in liquid nitrogen and processed for metabolome analysis. (JPG) [file pone.0252920.s001.jpg]
